# Revisiting the Relationships between Fat-to-Protein Ratio in Milk and Energy Balance in Dairy Cows of Different Parities, and at Different Stages of Lactation

**DOI:** 10.3390/ani11113256

**Published:** 2021-11-14

**Authors:** Edward H. Cabezas-Garcia, Alan W. Gordon, Finbar J. Mulligan, Conrad P. Ferris

**Affiliations:** 1 Estación IVITA—Maranganí, Universidad Nacional Mayor de San Marcos (UNMSM), Cusco 08258, Peru; 2 Sustainable Agri-Food Sciences Division, Agri-Food and Biosciences Institute (AFBI), Large Park, Hillsborough Co. Down BT26 6DR, UK; Conrad.Ferris@afbini.gov.uk; 3 Biometrics and Information Systems Branch, Agri-Food and Biosciences Institute (AFBI), AFBI Headquarters, 18a Newforge Lane, Belfast BT9 5PX, UK; Alan.Gordon@afbini.gov.uk; 4 School of Veterinary Medicine, University College Dublin, Belfield, D04 VIW8 Dublin, Ireland; finbar.mulligan@ucd.ie

**Keywords:** dairy cows, early lactation, energy balance, fat-to-protein ratio in milk

## Abstract

**Simple Summary:**

Data from 840 Holstein-Friesian cows (1321 lactations) were used to evaluate trends in fat-to-protein ratios in milk (FPR), and the use of FPR as an indicator of energy balance (EB). The fat-to-protein ratio was negatively related to EB, and this relationship became more negative with increased parity. Regression slopes describing linear relationships between FPR and EB differed over time, although trends were inconsistent. Similarly, ‘High’ FPR scores in milk (≥1.5) were consistently associated with a greater negative energy balance, milk yields, body weight loss, and plasma non-esterified fatty acid concentrations; however, their relationships with dry matter intake did not follow a clear trend. Although FPR can provide an indication of EB at a herd level, this analysis suggests that FPR cannot accurately predict the EB of individual cows.

**Abstract:**

A statistical re-assessment of aggregated individual cow data was conducted to examine trends in fat-to-protein ratio in milk (FPR), and relationships between FPR and energy balance (EB, MJ of ME/day) in Holstein-Friesian dairy cows of different parities, and at different stages of lactation. The data were collected from 27 long-term production trials conducted between 1996 and 2016 at the Agri-Food and Biosciences Institute (AFBI) in Hillsborough, Northern Ireland. In total, 1321 lactations (1 to 20 weeks in milk; WIM), derived from 840 individual cows fed mainly grass silage-based diets, were included in the analysis. The energy balance was calculated daily and then averaged weekly for statistical analyses. Data were further split in 4 wk. intervals, namely, 1–4, 5–8, 9–12, 13–16, and 17–20 WIM, and both partial correlations and linear regressions (mixed models) established between the mean FPR and EB during these periods. Three FPR score categories (‘Low’ FPR, <1.0; ‘Normal’ FPR, 1.0–1.5; ‘High’ FPR, >1.5) were adopted and the performance and EB indicators within each category were compared. As expected, multiparous cows experienced a greater negative EB compared to primiparous cows, due to their higher milk production relative to DMI. Relatively minor differences in milk fat and protein content resulted in large differences in FPR curves. Second lactation cows displayed the lowest weekly FPR, and this trend was aligned with smaller BW losses and lower concentrations of non-esterified fatty acids (NEFA) until at least 8 WIM. Partial correlations between FPR and EB were negative, and ‘greatest’ in early lactation (1–4 WIM; r = −0.38 on average), and gradually decreased as lactation progressed across all parities (17–20 WIM; r = −0.14 on average). With increasing parity, daily EB values tended to become more negative per unit of FPR. In primiparous cows, regression slopes between FPR and EB differed between 1–4 and 5–8 WIM (−54.6 vs. −47.5 MJ of ME/day), while differences in second lactation cows tended towards significance (−57.2 vs. −64.4 MJ of ME/day). Irrespective of the lactation number, after 9–12 WIM, there was a consistent trend for the slope of the linear relationships between FPR and EB to decrease as lactation progressed, with this likely reflecting the decreasing milk nutrient demands of the growing calf. The incidence of ‘High’ FPR scores was greatest during 1–4 WIM, and decreased as lactation progressed. ‘High’ FPR scores were associated with increased energy-corrected milk (ECM) yields across all parities and stages of lactation, and with smaller BW gains and increasing concentrations (log transformed) of blood metabolites (non-esterified fatty acid, NEFA; beta-hydroxybutyrate, BHB) until 8 WIM. Results from the present study highlight the strong relationships between FPR in milk, physiological changes, and EB profiles during early lactation. However, while FPR can provide an indication of EB at a herd level, the large cow-to-cow variation indicates that FPR cannot be used as a robust indicator of EB at an individual cow level.

## 1. Introduction

The energy balance (EB) in dairy cows can be defined as the difference between energy consumed from feed (input) and energy expended for maintenance, production, activity, and pregnancy (output). In early lactation, high-yielding dairy cows are often unable to consume sufficient nutrients to meet their energy requirements for milk production and, consequently, enter into a period of negative EB (NEB) [1], which in turn is reflected in the mobilization of body tissue reserves [2], primarily fat, as indicated by Komaragiri and Erdman [3]. Conversely, as the lactation progresses, cows normally move into a period of positive EB, and this is reflected in cows laying down body tissue reserves. The severity of NEB during early lactation has been linked to a number of problems. For example, NEB can delay the recovery of the postpartum reproductive function [4,5,6] and results in an altered immune response to pathogens (i.e., macrophage function), which may lead to health disorders such as mastitis and endometritis [7,8]. Therefore, understanding EB changes at both a herd level and at an individual cow level is important to allow farmers to take corrective actions before they manifest themselves in metabolic diseases, ill health, or poor fertility. 

A number of indicators have been used to provide an indirect assessment of EB, and to assist with the early detection of metabolic or management problems at a herd level. Changes in BW have been used as an indicator for EB, with the increasing availability of automated weighing systems on commercial farms making this approach more accessible [9,10]. However, caution must be exercised when using BW change data since they reflect both changes in tissue mass and gut fill. While the body condition score (BCS) assessment is another inexpensive and easy to implement technique to assess the body tissues reserves (and, by proxy, energy status) of lactating cows [11,12], it is still a subjective measurement and not sensitive enough to monitor changes in EB on a weekly basis [13]. However, with developments in automated image analysis systems, the automated assessment of BCS may become an increasingly useful tool for monitoring EB [12,14].

Blood metabolites, such as beta-hydroxy butyrate (BHB) and non-esterified fatty acids (NEFA) are also frequently advocated as indicators of the energy status in dairy cows, with their concentrations being related to lipid mobilization. Negative EB in early lactation is characterized by high concentrations of both NEFA and BHB [15], with threshold values for these metabolites having been established to predict clinical disease [16,17]. Nevertheless, the collection of blood samples is invasive, their analysis can be expensive, and repeated sampling is required to understand long-term patterns in EB. 

In contrast to blood, milk samples can be obtained daily during a normal milking routine, with milk composition known to change with the energy status [18,19]. Many factors influence milk yield and milk composition, including: (1) diet, for example, energy intake, forage to concentrate ratio, fat content of the diet, and, to a lesser extent, protein content and quality [20,21]; (2) body condition, whereby body stores can be mobilized for milk production during periods of NEB [3]; (3) milking frequency [22]; (4) many animal-related factors, including parity, age, breed, and stage of lactation [23]. Simple ratios between milk components, including the fat-to-protein ratio (FPR), have been proposed as indicators of EB during early lactation [18]. The biological basis of FPR as an indicator of EB relies on two tendencies: a mechanism that maintains the milk energy output by increasing the milk fat content when the yield is compromised due to a deficit in energy supply, and a decreased milk protein content under NEB [18]. The fat-to-protein ratio is known to be genetically negatively correlated to EB [24], and has gained considerable attention with studies indicating that FPR has a more robust relationship with EB in early lactation than individual measures of fat and protein content [25,26,27]. An FPR in milk between 1.0 and 1.5 has been considered ‘baseline’, representing normal physiological conditions in Holstein cows, whereas an FPR outside of this range has been associated with the occurrence of metabolic and health disorders, often during early lactation [28]. 

Frequent limitations of many studies, which have examined FPR as an indicator of EB, are the relatively small number of cows involved, and the use of EB proxies that may not provide a robust indicator of EB (e.g., blood metabolites). In addition, there is little information available on how effective FPR is as an indicator of EB for cows of different parities and at different stages of lactation. Therefore, the aim of the present study is to use a large dataset to explore the usefulness of FPR to act as an indicator for EB in Holstein-Friesian dairy cows offered predominantly grass silage-based diets, and to examine how changes in the FPR vs. EB relationship changes with the lactation number and within the stage of lactation during the first 20 weeks in milk (WIM). 

## 2. Materials and Methods

### 2.1. Experiments, Treatments and Cows

This study involved a statistical re-assessment of aggregated individual cow data obtained from 27 studies, which were conducted between 1996 and 2016 at the Agri-Food and Biosciences Institute (AFBI) in Hillsborough, Northern Ireland. The results of the majority of these studies have been published in peer-reviewed scientific papers, conference proceedings, technical reports, and a Ph.D. Thesis (Appendix A). A minimum prerequisite for the inclusion of any experiment in the analysis was that the experiment encompassed the ‘early lactation period’ (commencing within a few days of calving, and having a mean length of more than 90 days in milk; DIM), and included data on daily DMI, daily milk yield, regular milk composition, BW and BCS, and detailed information on diets offered (forage and concentrate composition and concentrate ingredients). The 27 experiments were variable in length, encompassing part lactations, full lactations, or multiple lactations. In the case of multi-lactation studies conducted over a number of years, each ‘year’ was designated as a separate experiment within the analysis. Within all experiments, the cows were transferred to a free stall cubicle house shortly after calving. Cubicles were fitted with rubber mats which were bedded with sawdust, while the house had concrete flooring which was scraped with an automated slurry scraping system. While some full lactation and multi-lactation experiments involved periods of grazing in mid/late lactation, data included in the analysis were restricted to periods when cows were housed and when individual cow feed intakes were available.

A total of 79 treatments were examined across the 27 studies, with the majority of treatments examining the impact of diet and/or management strategies on cow performance. While, a number of studies (*n* = 6) involved a comparison of two cow genotypes, only data from Holstein-Friesian cows were included within this analysis. In addition, individual cows within experiments were excluded from the analysis if they were on an experiment for less than the first 90 days’ post calving (or if the housed period when individual feed intakes were measured was less than 90 days). Data recorded during the first three days of lactation were excluded from the analysis, while data were included in the analysis up until a maximum of 140 DIM (provided individual feed intake data were recorded during the entire period). In addition, cows with a lactation number > 6 were excluded from the analysis. 

The final dataset represented 840 individual cows, and comprised data from 1321 individual lactations, 474 of which were primiparous, with the remainder multiparous (second to sixth lactations). At parturition, cows included in the present study had an average BW of 576 ± 76.9 kg, an average BCS of 2.57 ± 0.572, and an average parity of 2.4 ± 1.40. The relatively narrow range of BCS values reflected management practices adopted on the experimental farm. Pedigree records were available for the majority of the cows (*n* = 631), with these cows having a mean Predicted Transmitting Ability (PTA_2018_) for milk fat plus protein yield of 10 (S.D. 14.0) kg, and a mean Profitable Lifetime Index (PLI_2018_) of GBP 84 (S.D. 154.2). The conversion rate (British pounds to US dollars) at the time when genetic data were sourced was GBP 1 = USD 1.2652. 

### 2.2. Animal Measurements 

A number of animal measurement protocols changed over the 20-year period during which the 27 experiments were undertaken, while others remained largely unchanged. The feed intake of each individual cow was recorded daily using feed-boxes mounted on weigh cells, access to which was controlled by a Calan Gate feeding system (American Calan Inc., Northwood, NH, USA) linked to an electronic cow identification system. All diets were offered ad libitum. In all experiments, cows were milked twice daily, with milk yields recorded automatically at each milking, and a total daily milk yield for each cow determined for each 24 h period. In early experiments (*n* = 7), milk samples were taken in proportion to yield during six consecutive milkings (either weekly or fortnightly), and a single bulked sample analyzed. However, in later experiments (*n* = 20) samples were taken during two consecutive milkings (normally on a weekly basis) and each individual sample analyzed, and a weighted composition for the 24 h sampling period, subsequently, determined. Samples in all experiments were analyzed for fat, protein, and lactose concentrations (MilkoScan, Model FT 120, Foss UK Ltd., Warrington, UK), while milk somatic cell count (SCC) was determined monthly using a Fossomatic 360 (FOSS Electric, Hillerød, Denmark). Fat-to-protein ratio in milk was calculated as milk fat content (g/kg) divided by milk protein content (g/kg). 

The equation given by Tyrrell and Reid [29] was used to calculate the gross energy (GE) content of the milk (Equation (1)):

GE, MJ/kg= [0.0384 × fat] + [0.0223 × protein] + [0.0199 × lactose] − 0.108(1)

Energy-corrected milk yield (ECM; kg/day) was calculated assuming the GE content of 1 kg ‘standard milk’ to be 3.1 MJ/kg (i.e., for milk containing 4.0% fat, 3.2% crude protein, and 4.8% lactose, as described by Muñoz et al. [30], according to following equation (Equation (2)):

ECM, kg/day= (milk yield (kg/day) × GE (MJ/kg))/3.1(2)

Milk energy output (MJ/cow/day) was calculated by multiplying the GE content of milk (Equation (1)) by the daily milk yield. Feed efficiency was calculated by dividing ECM yield (kg/day) by the total DMI (kg/day). In early studies (*n* = 4), BW was recorded weekly, immediately after pm milking. However, in later studies, BW was recorded twice daily (immediately after each milking) using an automated weighbridge, and an average BW calculated for each week. Body condition scores were recorded weekly or fortnightly through each lactation, with BCS assessed by an experienced trained operator on a 1 to 5 scale [11]. Blood samples were collected (from the tail vein) in 26 of the 27 studies, normally between 8.00 and 10.00 h, while the frequency of blood sampling varied according to experiments (normally one sample every 14–28 days, until approximately week 12 WIM, with less frequent sampling thereafter). Blood serum was, subsequently, analyzed for BHB and NEFA concentrations.

### 2.3. Diets Offered and Determination of Energy Contents of the Feedstuffs

Although diets offered in these studies were predominantly based on grass silage and concentrates, diets changed over time, including concentrate feed levels and the ingredient composition of the concentrates offered. Grass silages offered were produced from perennial ryegrass (*Lolium perenne* sp.)-based swards, and were produced from primary growth and primary, secondary, and tertiary regrown herbages. Herbages were normally wilted prior to ensiling (typically for 24–48 h) and treated with a bacterial inoculant at harvesting. While silage offered in most studies was ensiled in clamp silos, a number of studies involved silage made in big bales (*n* = 4). In a number of studies (*n* = 16), grass silage was partially replaced with corn silage (usually between 20 and 40% of the forage component of the diet). In addition, in one study, a small quantity of chopped wheat straw (0.3 kg/cow/day) was included in the diet. In all studies, the forage component of the diet was offered *ad libitum* (normally, between 7 and 10% of the previous day’s intake). 

A wide range of concentrate types, feeding levels, feeding strategies, and feeding methodologies were adopted within and between studies, according to the objectives of each individual study. The concentrate supplements consisted, principally, of cereal grains (e.g., barley, wheat, corn), protein supplements (e.g., soybean meal, canola meal), and by-products from the food industry (e.g., corn gluten meal, sugar-beet pulp, citrus pulp). Additional energy sources (e.g., Megalac^®^ and molasses) were included in some concentrates, while most concentrates contained a mineral/vitamin supplement. The concentrate component of the diets was offered either mixed with the forages (partial mixed ration), separate from the forages (via in-parlor or out-of-parlor feeders), or via a combination of these practices. The mean forage: concentrate DM ratio across the 27 studies was 49:51.

In all experiments, samples of grass and corn silages offered were collected daily for oven dry matter (ODM) analysis, with fresh samples normally analyzed weekly for nitrogen (N), gross energy (GE) and fermentation products. Silage ODM contents were, subsequently, corrected for volatile losses during drying, with all intakes presented on a volatile corrected DM basis. Samples of dried silage, composited for each 2–4-week period, were, subsequently, analyzed for fiber and ash concentrations. 

In early experiments (*n* = 8), the digestible OM in total DM content (DOMD, %) of silages was determined by offering the silages to sheep confined in ‘digestibility crates’ at maintenance level (normally 4 sheep per silage). The metabolizable energy (ME) content of these silages was then estimated by multiplying the DOMD by 0.16 (assuming that one percentage point of DOMD equates to 0.16 MJ/kg DM of ME) [31]. The calculated ME values were then corrected to ‘production level of feeding’ by multiplying by 0.97 [32,33]. In later experiments (*n* = 20), the ME value of the forages offered was derived using NIRS as described by Park et al. [34]. In two experiments, where neither sheep digestibility data nor NIRS predictions were available, silage DOMD was initially predicted from nutrients composition (DM, Ash, CP, and NDF) and fermentation characteristics of the silages (lactic acid: total VFA ratio) as described by Yan and Agnew [35] (Equation (14b)), and silage ME content estimated by multiplying the DOMD by 0.16. The mean ME content of the silages offered was 11.3 ± 0.58 for grass silages and 11.2 ± 0.35 for corn silages, while the ME content of wheat straw was assumed to be 6.0 MJ/kg DM (FeedByte^®^, SRUC, Edinburgh, UK).

Concentrates offered were normally sampled weekly, and composite samples analyzed for each 2–4 wk. period. The ME content of each concentrate was calculated using the ME content of each individual ingredient, based on values reported in UK feed composition tables (FeedByte^®^). The mean calculated ME content of the concentrate offered was 12.9 ± 0.25 MJ/kg DM. Total ME intakes were determined as the sum of the DM intake of each diet component multiplied by the ME content of that component. Further details of analytical methods used to determine the chemical composition of the feedstuffs and fermentation quality of the silages are presented within the individual studies, which are listed in Appendix A.

### 2.4. Calculations of Estimated EB

Individual cow EB values were initially calculated on a daily basis. Daily EB calculations utilized daily DMI and daily milk yield values. However, for data that were not available on a daily basis (i.e., BW and milk composition data), measured values were applied to each day during the 3-day period pre and post the day of measurement (in the case of weekly measurements), or to the 7-day period pre, and to the 6-day period post the day of measurement (in the case of fortnightly measurements). The mean ME content of all individual silage samples taken from each silo was applied to all days during which that silage was offered.

The daily estimated EB (EB, MJ of ME/day) of each individual cow was calculated using equations contained within ‘Feed into Milk’ (FiM), the current UK dairy cow rationing system, as the difference between the cow’s total ME requirements (maintenance, milk production, and activity) and total ME intake [36]. The sum of ME requirements for maintenance, activity (included within maintenance component, includes standing, vertical movement, and body position changes) and milk production (ME_maint+milk_: MJ/kg of BW^0.75^) was determined using Equation (3).

ME_maint+milk_ = log_e_ [[[5.06 − Milk E. per kg of BW^0.75^]/[5.06 + 0.453]]])/ − 0.1326 (3)

where Milk E. = milk energy (MJ). Pregnancy requirements were excluded from all EB calculations in the present study since data used within this analysis were until a maximum of 140 DIM, a 42-day voluntary waiting period was adopted within the AFBI herd, and FiM pregnancy was only accounted for from week 14 of gestation onwards. Energy requirements for ‘walking’ were included within the EB calculations as described by Agnew et al. [36] (shown in Equation (4)), using the term (0.0013 × BW)/*k_m_*, with the efficiency of utilization of ME for walking assumed to be the same as that for maintenance (*k_m_* = 0.35 × ME/gross energy + 0.503) [31]. This assumes a distance walked of 500 m, which was considered appropriate for housed cows on the AFBI farm. Finally, the estimated daily EB for each individual cow was calculated using the following equation:

EB, MJ of ME/day = ([ME_maint+milk_ × BW^0.75^] + [[0.0013 × BW] /*k_m_*] − 10) − ME_i_ (4)

The term ME_i_ is the ME intake (MJ/cow per day). Mean weekly EB values were, subsequently, calculated for each week post-calving (up to a maximum of 20 WIM), with calving date considered as day 1 of the first WIM.

### 2.5. Statistical Analysis

The full dataset comprised 25,605 cow-week observations, with the individual cow lactation considered as the experimental unit. Individual cows included in the dataset were not fully nested within a study since individual cows were often used in more than one study. The dataset was subdivided by lactation number as follows: lactation 1, lactation 2, lactation 3, and lactation ≥ 4, the latter comprising cows in lactations 4, 5, and 6. Least mean squares evaluating the interaction between lactation number (1, 2, 3, and ≥4) and WIM were calculated to obtain general trends for DMI, ECM, EB, BW and BCS, milk composition parameters, and blood metabolites (BHB and NEFA). Both study and cow were included as random crossed effects in the model, and differences between least square means tested with the Tukey–Kramer test when relevant. 

Partial correlations between FPR and EB were determined using the MANOVA option of PROC GLM of SAS (version 9.4; SAS Institute Inc. Cary, NC, USA) to obtain partial correlations controlling for the effect of individual study within the dataset, at five 4 wk. intervals, namely, 1–4, 5–8, 9–12, 13–16, and 17–20 WIM. Univariate linear mixed model regressions were built to evaluate FPR as a single predictor of EB at the same time intervals. The best fit to the equation was chosen based on the lowest root mean square error (RMSE). All regressions analyses were performed within the MIXED procedure of SAS [37], and the REML method (restricted maximum likelihood) statement was used in the PROC MIXED model syntax. In the RANDOM statement, TYPE = VC was used as an option as variance–covariance structure, with study and cow included as random crossed effects in the model. The slopes of regressions between each of the 5 WIM intervals within each lactation of the independent variable (FPR) were pairwise compared by T-test. 

Three FPR score categories (‘Low’ FPR, <1.0; ‘Normal’ FPR, 1.0–1.5; ‘High’ FPR, >1.5) were adopted, and the PROC FREQ procedure of SAS used to determine the percentage occurrence of each FPR score during each four wk. interval from calving until 20 WIM. Prior to analysis, a natural logarithm transformation was applied to NEFA (lnNEFA) and BHB (lnBHB) concentrations to achieve normality. Animal performance (DMI, ECM), body reserves (BW change), log-transformed blood metabolites (lnBHB and lnNEFA), and daily EB associated with each FPR scores were assessed at selected four-week intervals using the following statistical model:

Y_ijk_ = μ + F_i_ + S_j_ + C_k_ + E_ijk_ (5)

where Y_ijk_ is a dependent variable, μ is the mean for all observations, F_i_ is the effect of FPR score i, S_j_ is the effect of study j, C_k_ is the effect of cow k, and E_ijk_ ~N(0, σ2e) represents the residual error. Both study and cow were included as random crossed effects in the model and differences between least square means tested by pairwise comparisons with the Tukey–Kramer test when relevant. Denominator degrees of freedom were calculated by the Kenward–Roger adjustment [38]. For all models, statistical significance was declared at *p* ≤ 0.05.

## 3. Results

### 3.1. Animal Performance Trends during Early Lactation

**Figure 1:**
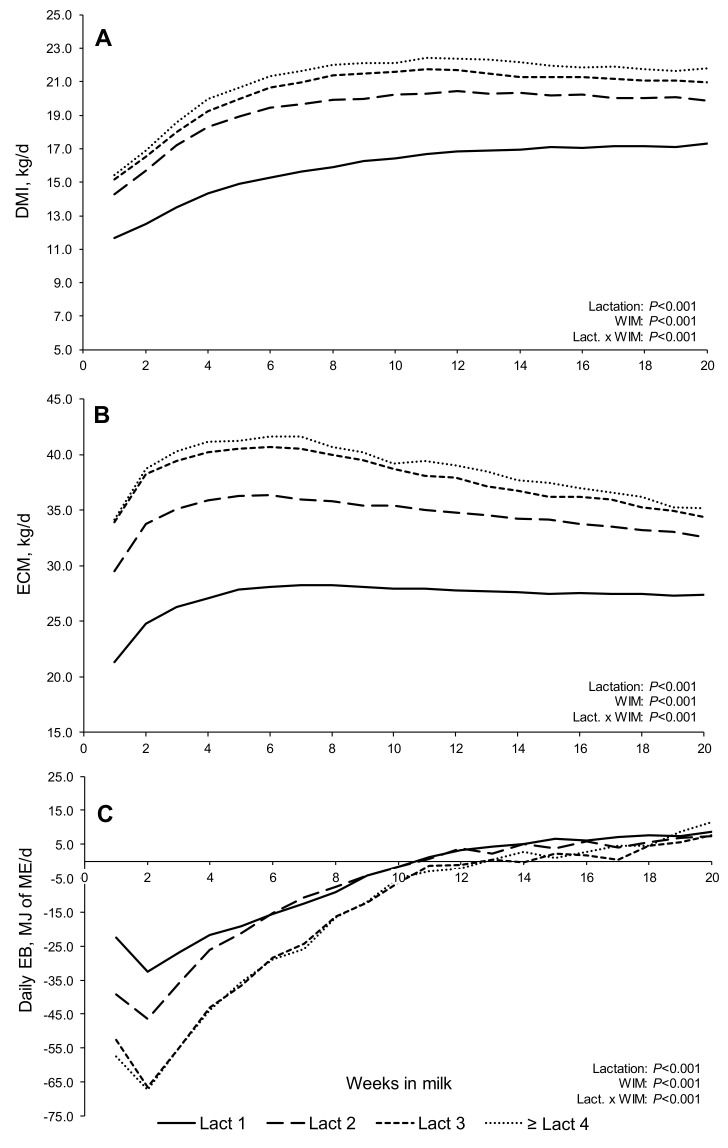
Least squares means, adjusted by the random crossed effects of study and cow, for dry matter intake (DMI; (**A**)), energy-corrected milk (ECM; (**B**)), and estimated energy balance (daily EB; (**C**)) according to equations within ‘Feed into Milk’ [36], in early lactation dairy cows (1–20 WIM).

**Figure 2:**
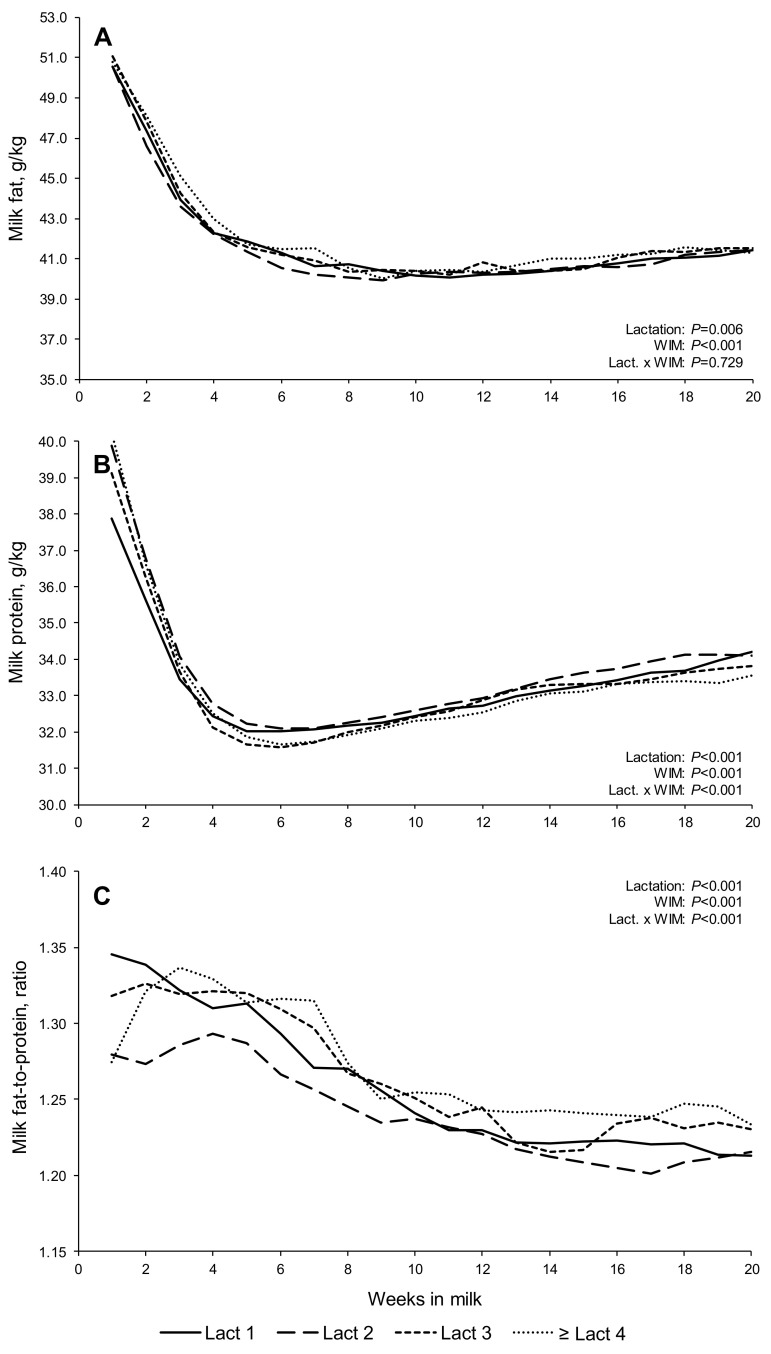
Least squares means, adjusted by the random crossed effects of study and cow, for milk composition variables—milk fat (**A**); milk protein (**B**); and milk fat-to protein ratio (**C**)—in early lactation dairy cows (1–20 WIM).

In general, trends across lactations for the main parameters (Table 1) were as expected, with the mean DMI, milk production, and BW increasing with the lactation number. Multiparous cows displayed a higher energy deficit than primiparous cows (greater NEB), due to their higher milk production relative to DMI. The relatively narrow range in BCS observed within lactations was likely due to management practices within the AFBI herd. Weekly trends for DMI, ECM, EB (Figure 1A–C, respectively), and milk solids contents (fat, protein, and FPR; Figure 2A–C, respectively) were presented for the first 20 WIM for cows in lactations one, two, three, and ≥ four. All parameters differed between lactations (DMI, ECM EB, milk protein content, FPR, *p* < 0.001; milk fat content *p* < 0.006) and with the stage of lactation (*p* < 0.001), while there was a significant lactation × WIM interaction for all parameters (DMI, ECM, EB, milk protein content, *p* < 0.001; FPR, *p* = 0.001), with the exception of milk fat content (*p* > 0.10). As expected, peaks in the ECM yield across lactations occurred earlier compared to responses in DMI (Figure 1A,B). Irrespective of the lactation number, nadir EB occurred around the second WIM (Figure 1C), and became more negative with increasing parity (−32.5, −46.4, and −66.4 MJ of ME/day for lactations one, two, and three, respectively; *p* < 0.001). No significant difference was detected for the nadir point between lactations three and ≥ four. Primiparous and second lactation cows reached positive EB earlier (≈10.5 WIM) compared to third and ≥ fourth lactations (≈12.5 WIM), respectively. Dry matter intakes reached a plateau (95% of maximum observed intake) at 10 and 6 WIM in primiparous and multiparous cows, respectively, while the peak ECM (maximum production measured as weekly least square means) occurred at 6 WIM in all lactations (peak yields of 28.2, 36.3, 40.6, and 41.6 kg/day for lactation one, two, three, and ≥ four, respectively).

Concentrations of milk fat declined from the beginning of lactation until, approximately, 8 WIM, remaining relatively constant thereafter (Figure 2A). In contrast, while milk protein concentrations declined rapidly until approximately 6 WIM, concentrations increased gradually thereafter (Figure 2B). At the first WIM, primiparous cows displayed lower protein contents compared to multiparous cows (37.5 vs. 39.8 g/kg on average). In general, FPR curves tended to decrease as the lactation progressed, with second lactation cows tending to have the lowest FPR values throughout the 20 WIM period (Figure 2C).

**Figure 3:**
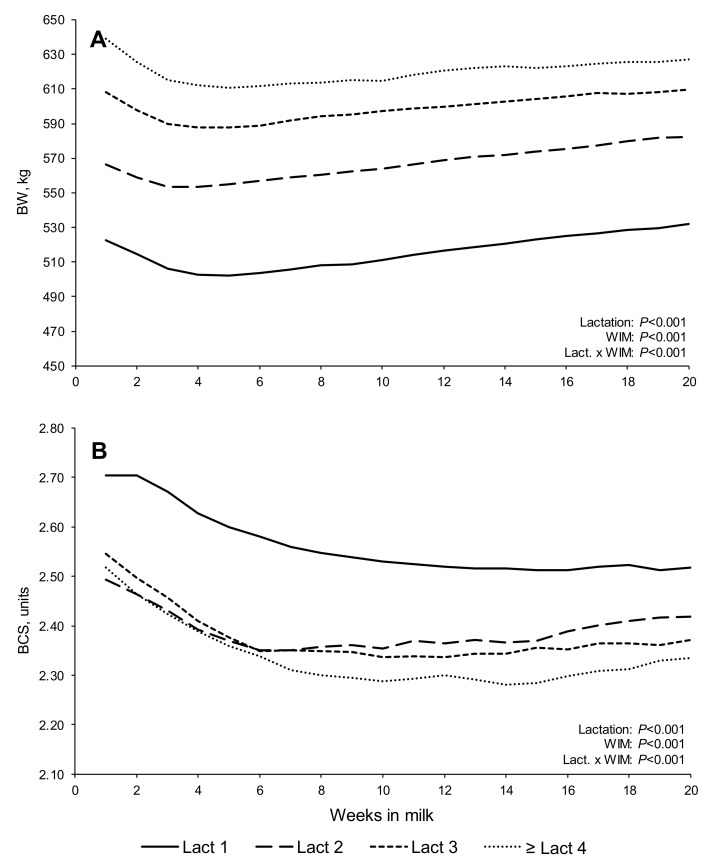
Least squares means, adjusted by the random crossed effects of study and cow, for body tissues reserves variables—body weight (BW; (**A**)); and body condition score (BCS; (**B**))—in early lactation dairy cows (1–20 WIM).

**Figure 4:**
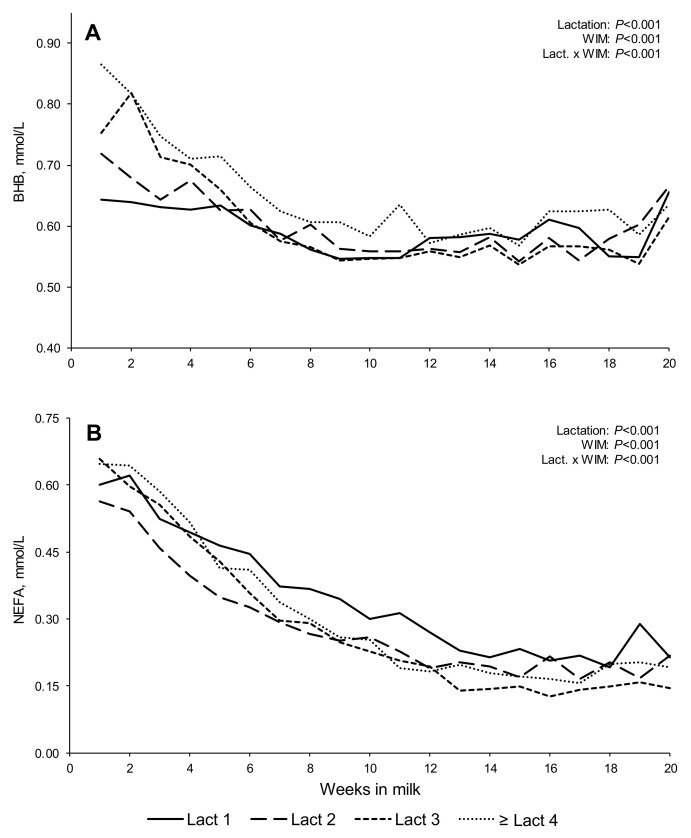
Least squares means, adjusted by the random crossed effects of study and cow, for blood metabolites—beta-hydroxybutyrate (BHB; (**A**)), and non-esterified fatty acids (NEFA; (**B**))—in early lactation dairy cows (1–20 WIM).

Changes in body tissues (BW and BCS) and blood metabolites (BHB and NEFA) are presented in Figure 3 and Figure 4, respectively. All of these variables differed with lactation and WIM (*p* > 0.001). With all parities, the cows tended to lose BW during the first few WIM, gaining BW thereafter, while the BCS decreased throughout most of the first 20 WIM in primiparous cows, while, with multiparous cows, BCS decreased until 6–14 WIM (depending on lactation number), with cows tending to slowly gain BCS thereafter. Multiparous cows had higher BHB concentrations in early lactation than primiparous cows, with concentrations decreasing until approximately 8 WIM, remaining relatively constant thereafter. Non-esterified fatty acids concentrations tended to decrease until, approximately, 12–14 WIM, remaining relatively constant thereafter, with second lactation cows tending to have lower NEFA concentrations until, approximately, 7 WIM. Maximum plasma concentrations of both BHB and NEFA were observed during the first 2 WIM.

### 3.2. Animal Performance Trends during Early Lactation

**Figure A1:**
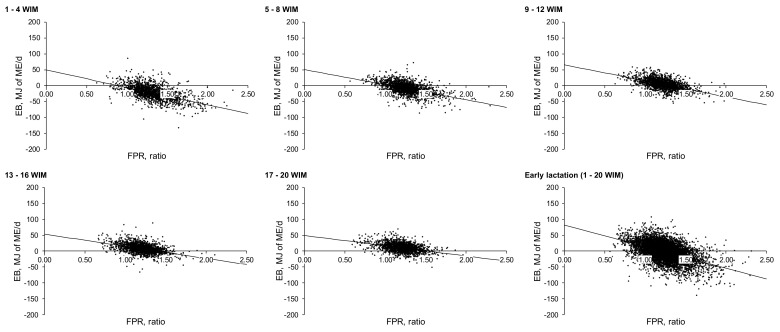
Linear relationships between fat-to-protein ratio in milk (FPR) and daily energy balance (EB, MJ of ME/day) in first lactation Holstein-Friesian dairy cows (1–20 WIM). Mixed regression models for each relationship are presented in Table 3.

**Figure A2:**
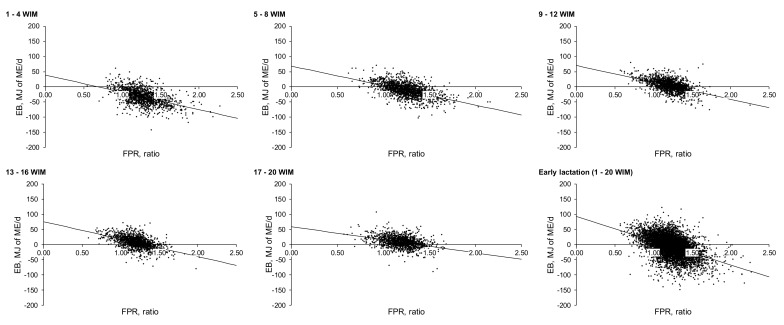
Linear relationships between fat-to-protein ratio in milk (FPR) and daily energy balance (EB, MJ of ME/day) in second lactation Holstein-Friesian dairy cows (1–20 WIM). Mixed regression models for each relationship are presented in Table 3.

**Figure A3:**
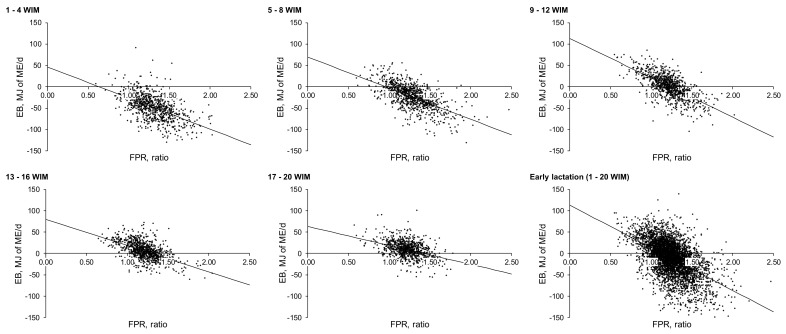
Linear relationships between fat-to-protein ratio in milk (FPR) and daily energy balance (EB, MJ of ME/day) in third lactation Holstein-Friesian dairy cows (1–20 WIM). Mixed regression models for each relationship are presented in Table 3.

**Figure A4:**
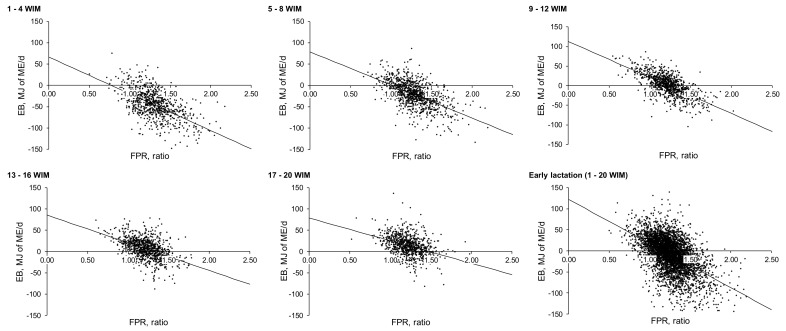
Linear relationships between fat-to-protein ratio in milk (FPR) and daily energy balance (EB, MJ of ME/day) in ≥ fourth lactation Holstein-Friesian dairy cows (1–20 WIM). Mixed regression models for each relationship are presented in Table 3.

When examined across 1–20 WIM, FPR was moderate and negatively correlated (*p* < 0.001) with EB (r= −0.334, −0.282, −0.354, and −0.347 for lactations one, two, three, and ≥ four, respectively), while within each of lactations 1–4, partial correlations between these two variables tended to become less negative with an increased stage of lactation (Table 2). All linear mixed models developed between FPR and EB (at four wk. intervals post calving, and for the entire 20 WIM period, within each of lactations one, two, three, and ≥ four; Table 3), were significant (*p* < 0.001), with FPR consistently negatively related to EB. The data used to develop each of the models in Table 3 were presented for each time interval within each lactation as Figure A1, Figure A2, Figure A3 and Figure A4 (Appendix B). 

### 3.3. Fat-to-Protein Ratio Scores and Their Associations with EB Indicators

**Figure 5:**
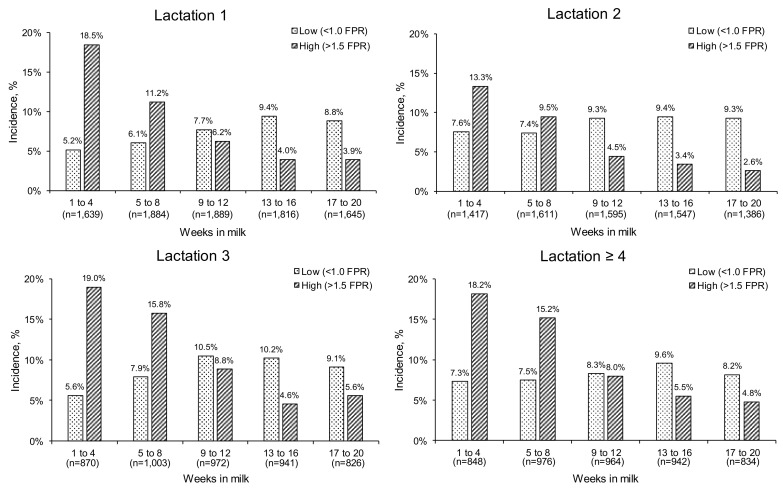
Incidence of extreme fat-to-protein ratio scores in milk (FPR scores: <1.0 = ‘Low’, and >1.5 = ‘High’) across lactations (1, 2, 3, and ≥4) at specific at four WIM intervals in early lactation dairy cows (1–20 WIM). The number of observations (*n*) include ‘Normal’ values (1.0–1.5 FPR).

When examined during each four-week interval during each lactation, the incidence (%) of ‘High’ FPR scores (>1.5) tended to decrease as each lactation progressed, whereas, in general, the incidence of ‘Low’ scores (<1.0) tended to increase until 9–12 WIM, remaining relatively constant thereafter (Figure 5). Least squares means for DMI, ECM, milk fat and milk protein content, BW changes, lnBHB, lnNEFA, and EB were presented for cows with ‘Low’, ‘Normal’, and ‘High’ FPR (at 4 wk intervals over 1–20 WIM) in Table 4, Table 5, Table 6 and Table 7 (lactations one, two, three, and ≥ four, respectively). The ‘High’ FPR scores were often, but not always, associated with decreasing DMI, while increasing FPR values were related to an increase in ECM, irrespective of parity and the stage of lactation (*p* < 0.001). Similarly, across all parities and lactation intervals, increasing FPR scores were associated with increasing milk fat contents and decreasing milk protein contents (*p* < 0.009). Increased FPR was often associated with a greater BW loss or smaller BW gain, and increasing lnBHB and lnNEFA concentrations, with these effects mostly significant during 1–4 and 5–8 WIM post calving. Finally, ‘High’ FPR scores were always associated with a decrease in EB, irrespective of parity and the stage of lactation (*p* < 0.001).

## 4. Discussion

Data analyzed within this paper were derived from a single farm. This reflected the fact that the determination of daily EB values relied on the use of individual cow intake data, which are generally only recorded on research farms. However, a wide range of diet types and qualities were examined within the studies used in the analysis. Furthermore, while the genetic potential of cows in the research herd changed over time, this genetic variability within the dataset made the results highly applicable to commercial farms where genetic variation both within herds and between herds can be substantial.

In addition to characterizing the relationships between FPR and EB during the first 20 WIM, this paper sought to examine how the main drivers of EB (DMI and ECM) and other commonly used EB indicators (BW changes, BHB, and NEFA) might help explain FPR–EB relationships across the stage of lactation and parity in Holstein-Friesian dairy cows. The mean forage-to-concentrate proportion in diets offered across the 27 experiments was 49:51 (on a DM basis), with this ratio varying little across lactations; thus, minimizing possible confounding effects arising from differences in energy supply. This concentrate proportion in the diet is typical of that in diets offered to moderate–high-yielding dairy herds within grass silage-based systems in the UK.

### 4.1. Trends in Intakes, Milk Production, Blood Metabolites, FPR, and EB

An examination of weekly trends in DMI, milk production, milk composition, EB, BW, BCS, and blood metabolites allowed changes in FPR to be placed within a wider context. The trend for DMI and ECM to increase from lactations one to three within the lactation profiles reflected the increasing size (reflected in increasing BW) of the cows with increasing parity, their correspondingly greater intake and milk production potential, and their increased capacity for tissue mobilization in support of lactation, resulting in an increased duration of NEB [23]. In support of this, in the present study, both first and second lactation cows returned to positive EB, approximately, two weeks earlier than cows in lactations three and ≥ four (on average at 10.5 vs. 12.5 WIM, respectively). Furthermore, during the first lactation especially, cows are still growing and must partition a certain proportion of nutrients consumed to growth, as well as milk production, often resulting in lighter calves [39].

While the EB curves in the present study were based on weekly least square means, these curves were in agreement with modelled EB curves (polynomial regressions) for Holstein cows offered grass silage-based diets [27,40]. Although no differences were observed in the timing of nadir EB across lactations, nadir EB (MJ of ME/day) became more negative with parity. This was in agreement with earlier studies with nadir EB calculated by difference (energy intake minus energy expenditure) [40,41,42], and also when it was calculated from EB curves based on body energy changes [9,41,43]. In general, lactation curves increase to a peak as the cow seeks to meet the increasing nutrient needs of the calf, prior to the transition to a ‘solids’ diet, and the slow process of enforced weaning by the dam [39,44]. This corresponds with cows reaching the point of nadir EB in early lactation.

The milk fat and milk protein content followed normal lactation trends (providing the highest quality milk to the growing calf at a time when the calf was completely dependent on milk for growth), but with subtle differences between lactations and over time, which influenced FPR. The FPR ‘curves’ in the present study, which decreased from maximum values in early lactation and ‘plateaued’ around 10–13 WIM, were broadly similar to curves observed with Holstein cows in Germany [27], Canada [45], and Poland [46]. For example, in the mean FPR curves presented by Satoła and Ptak [46], and in the modeled FPR curves presented by Buttchereit et al. [27], the maximum FPR occurred during the second WIM, irrespective of parity. Nevertheless, a visual examination of the FPR curves in the latter study revealed a high degree of variability during the first month of lactation, with this variation being also evident during early lactation in the present study, the time when energy deficit was most pronounced. The FPR in the present study mirrored the trend observed in EB, suggesting a relationship between these two parameters. This also agreed with the ‘inverted patterns’ observed by Buttchereit et al. [27], where FPR and EB were modelled using polynomial regressions.

Nevertheless, across published studies, there is much variation in the ratio at which the curves plateaued, with curves plateauing at, approximately, 1.20 to 1.25 in the present study, between 1.05 (first and second lactation) and 1.15 (third and further lactation) in the study by Buttchereit et al. [21], and 1.15 in the study by Satoła and Ptak [46]. It is possible that this was due to genetic differences in the fat and protein content of the milk produced between cows in these studies. There were no obvious reasons for the trend towards a lower FPR curve with lactation two cows, although a close examination of the curves in Figure 2 demonstrated that relatively minor differences in milk composition at a given point in time could result in quite substantial differences in FPR.

That cows in all lactations started to gain BW from approximately 4 WIM onwards, long before reaching positive EB, has been observed in earlier studies [47,48], with this likely due in part to meal-related ‘gut-fill’ [9]. However, losses of BCS continued until, approximately, 6–10 WIM in multiparous cows and 12–14 WIM in primiparous cows; thus, following broadly similar, but mirror image, patterns to the FPR curves. In addition, Holstein dairy cows mobilize significant quantities of internal body fat reserves in early lactation, but this is not always fully reflected in BCS changes [49,50]. In the present study, the relatively modest degree of tissue mobilization (BW and BCS losses) might be partly related to the moderate BCS observed following calving (2.70, 2.49, 2.54, and 2.52 for lactations one, two, three, and ≥ four, respectively). Cows that have a higher BCS at parturition tend to lose more BCS postpartum than those with a low–moderate BCS [12,51,52].

The decreasing concentrations of BHB and NEFA in blood plasma, both of which are known to be indicators of body fat mobilization [15,16,53], were broadly aligned with the longitudinal weekly trends in BCS across lactations, although BHB concentrations appear to plateau earlier than NEFA concentrations. Maximum concentrations of BHB and NEFA during the first few WIM supported the occurrence of nadir EB within the second WIM for all lactations. The trend for lower weekly NEFA values early in lactation two, compared to other lactations, aligned with the trends in FPR curves and body tissue losses as described earlier.

### 4.2. Changes in FPR–EB Relationships with Stage of Lactation and Lactation Number

Uniquely, the present study allowed the relationships between FPR and EB to be examined at four wk. intervals during the first 20 WIM, and within each of lactations one, two, three, and > four. Irrespective of the lactation stage and parity, the partial correlations demonstrated that FPR was negatively related to EB, with the range of correlations observed broadly in agreement with those reported by Buttchereit et al. [27] for primiparous cows (r = −0.15 to −0.43) and for multiparous cows by Grieve et al. [25] (r = −0.36 to −0.74) and Reist et al. [54] (r = 0.50), respectively. In the study by Buttchereit et al. [27], phenotypical correlations between FPR and EB decreased as the lactation progressed (r = −0.42, −0.28, −0.20, at 35, 55, and 75 DIM, respectively), in agreement with genotypic correlations found later by the same authors [55], and in a more recent study by Harder et al. [56]. Thus, based on these correlations, FPR appears to be a stronger indicator of EB at the beginning of lactation when the cow experiences the greatest metabolic stress.

To the best of our knowledge, no previous study has examined FPR–EB relationships using a Linear Mixed Model approach evaluating between and within lactation responses as shown in the present study. The slopes of these regression models provide an estimate of the rate of decrease in EB per unit of increase in FPR. In the present study, when data from all 20 WIM were combined (slopes for the entire 20 WIM period for each lactation), the slope of the regression in each of lactations one to ≥ four was considerably more negative than for any of the individual four WIM periods within that lactation. An examination of the Figure A1, Figure A2, Figure A3 and Figure A4 (Appendix B) highlights that this was caused by the vertical ‘stacking’ of data clusters from each four wk. period, creating an apparent response line that was very different from responses within individual four WIM periods. This demonstrates the danger of combining data for multiple weeks (1–20 WIM) for a parameter (EB) which changes over time as shown in the EB curves. Nevertheless, the examination of Figure 1C, and Figure A1, Figure A2, Figure A3 and Figure A4 (Appendix B), suggests that the increase in the slope with increasing parity (largely consistent when data from each four wks. period were compared across parity) was not primarily due to a greater range in FPR with increasing parity, but rather a greater range in EB values.

When data were compared across time points within any lactation, the Figure A1, Figure A2, Figure A3 and Figure A4 demonstrated that, in general, the data points showed a general ‘contraction’ from the ‘right’ to the ‘left’ as lactation progressed (i.e., a decreasing number of FPR points > 1.5). In addition, the overall dataset shifted upwards as EB increased, while the range of EB values encountered also appeared to decrease with the stage of lactation. However, the overall trends in slopes within each lactation were inconsistent, with the most negative slope occurring at 1– 4 WIM in lactations one and ≥ four, and at the WIM intervals of 5–8, 9–12 in lactations two and three, respectively. Reasons for the inconsistent trends across lactations are unclear, and our findings contrasted with the findings of Buttchereit et al. [27], based on polynomial regressions of relationships between FPR and EB, that, irrespective of parity, the most NEB responses per unit of FPR were observed only within the first month of lactation.

The data presented in Appendix B clearly highlight the wide range in individual cow FPR values, something that was not reflected in the relatively narrow range in mean values presented in Figure 2C. Furthermore, the linear models suggest that the interaction between the stage of lactation and parity would need to be considered if attempting to use FPR as a single predictor (indicator) of EB in dairy cows during early lactation. Earlier studies indicated that differences in genetic correlations between FPR and EB as lactation progressed may be due to different genes regulating physiological mechanisms affecting EB at different times during early lactation [42,55,57,58].

### 4.3. Performance Indicators Associated with ‘Low’ and ‘High’ FPR Scores

While a ‘baseline’ FPR score between 1.0 and 1.5 is considered to represent normal physiological conditions in Holstein cows during early lactation [59], an FPR outside of this range has been associated with the occurrence of metabolic and health disorders during early lactation. In particular, cows with an FPR greater > 1.5 are associated with an increased risk of ketosis, displaced abomasum, ovarian cysts, lameness, and mastitis [24,28,59,60], while those with an FPR of less than 1.0 have been found to have an increased risk of sub-acute ruminal acidosis (SARA) [61,62]. In the present study, the incidence of ‘High’ FPR scores decreased as lactation progressed (17.2, 12.9, 6.9, 4.4, and 4.2% at 1–4, 5–8, 9–12, 13–16, and 17–20 WIM, respectively; mean data across all lactations). A similar trend was observed in the study by Vlček et al. [63], where the frequency of ‘High’ FPR scores (ketosis risk; >1.5 FPR) of 908 test-day records (208 Holstein cows) was found to be slightly higher during the first month of lactation (≈22%) and similar decreases observed with advanced lactation (≈10, 8, 4, and 3% for the second, third, fourth, and fifth month of lactation, respectively). In contrast, there was a small increase in the incidence of ‘Low’ FPR scores (<1.0) as lactation progressed (6.4, 7.2, 9.0, 9.7, and 8.8% at 1–4, 5–8, 9–12, 13–16, and 17–20 WIM, respectively; mean data across all lactations), with the incidence tending to reach a plateau around the time cows returned to positive EB. In contrast, in the study by Vlček et al. [63], a more pronounced shift towards a greater incidence of ‘Low’ FPR scores (<1.0; risk of SARA) as lactation progressed was observed (≈11.0, 16.0, 19.0, 24.0, and 27.0% of cows at months 1–5 of lactations, respectively). Although the composition of diets offered in the latter study was not reported, these frequencies might suggest that the mean concentrate proportion in the diet offered may have been higher than in the present study. It is well known that an increased energy supply (via highly fermentable carbohydrates) in the diet of dairy cows is related to the increased risk of SARA, and this is reflected in low FPR values in milk [64,65].

The results presented in Table 4, Table 5, Table 6 and Table 7 allow an examination of how performance and blood parameters associated with each FPR score differed, with most of the traits presented either drivers of, or indicators of, EB. Across all parities and across all stages of lactation, EB showed a consistent decrease with an increasing FPR score, cows with an FPR score > 1.5 always having a significantly lower EB than those with an FPR score < 1.0. Both DMI and ECM are considered as primary factors influencing EB responses in dairy cows during early lactation, although their individual importance may vary according to parity and the stage of lactation. For example, studies by Hüttmann et al. [58] and Krattenmacher et al. [66] reported that EB was more genetically correlated to DMI than ECM across the first 180 DIM in primiparous cows. In contrast, Buttchereit et al. [24] reported that genetic correlations between DMI and EB only became more important than those for ECM and EB during mid lactation (≥120 DIM). Within the present study, differences in EB between scores were due in part to differences in DMI, with a trend, although not always consistent, for cows with ‘High’ FPR scores to have lower DMI. In contrast, ECM appeared to be a more consistent driver of differences in EB between FPR scores, increasing significantly across each of the three FPR score bands during every 4 wk. period in all parities. Trends for decreased milk production associated with ‘Low’ FPR scores in the present study were in good agreement with results presented by Toni et al. [28], who evaluated FPR at 7 DIM (test-day).

In agreement with the differences in EB between FPR scores, cows with an FPR score > 1.5 had a consistently greater BW loss, or lower BW gain, than cows with an FPR score < 1.0 throughout the first 12 WIM in all parities (except for 9–12 WIM in lactation ≥ four). In contrast, there were few differences in actual BCS between FPR score groups, and while this does not imply that changes in BCS did not follow a similar pattern for BW changes, it does suggest that cows with a different BCS were not more likely to be in one FPR score band than in another. This is perhaps surprising as there is ample evidence that cows with a high BCS are likely to have lower DMI and, subsequently, greater tissue loss in early lactation [12,49]. However, the present study has shown that lower DMI was not a consistent driver of NEB of cows having a ‘High’ FPR score. Despite this, Løvendahl et al. [67] found that EB estimated from milk composition variables generally follows a time profile similar to EB estimated from BCS and BW.

The increasing concentrations of both BHB and NEFA (ln transformed) in blood metabolites at higher FPR scores in early lactation reflected the greater NEB and greater BW loss with higher FPR scores. These metabolites, produced from the mobilization of body tissue reserves to produce glycerol for use as an energy resource, are normally associated with high milk fat concentrations and, as such, ‘High’ FPR scores (>1.5) [68]. Greater concentrations of each of these metabolites have been associated with an increased risk of subclinical ketosis, with risk thresholds for both plasma BHB and NEFA concentrations identified [16,17,69]. For example, a NEFA threshold of 0.57 mEq/L was identified by Ospina et al. [16] within the first 30 DIM for subclinical ketosis in North America. Untransformed NEFA concentrations in the present study at 1–4 WIM were 0.60, 0.52, 0.67, 0.68 mEq/L for lactations one, two, three, and ≥ four, respectively, greater than the threshold identified by Ospina et al. [16] in all parities, except for the second lactation cows. However, none of these mean values were higher than 0.70 mEq/L during the first 20 DIM, a value recently identified as being indicative of excessive NEB in commercial dairy herds in the UK [17]. Although BHB has been used as an indicator of NEB, high blood butyrate concentrations can reflect the rumen production of butyrate associated with an increased concentrate proportion in the diet [70]. In contrast, NEFA is less influenced by diet and is considered as a better indicator of adipose tissue lipolysis [10,71]. Concentrations of NEFA and BHB have been reported not to be well correlated during the transition period [72], which may partly explain discrepancies between these metabolites among FPR scores in the present study. The lowest incidences of ‘High’ FPR values observed with second lactation cows, especially during the first two WIM intervals, aligned with the smaller BW losses and lower NEFA concentrations when compared to cows in other lactations. Although data from the pre-partum period were not available in the present study, a smaller loss of BCS in second lactation cows during the entire transition period (including 3 wks. after parturition), compared to the other lactation groups, could partly explain these trends [73].

While this paper established clear relationships between FPR and EB, it is important to examine the utility of FPR as a prediction tool of EB for individual cows. Partial correlations and linear regression models highlighted strong relationships between EB and FPR, while data presented in Table 4, Table 5, Table 6 and Table 7 show that cows within each FPR score differed in performance and EB. Nevertheless, a visual examination of individual cow data in the Figure A1, Figure A2, Figure A3 and Figure A4 (Appendix B) did not suggest that FPR could be used to accurately predict the EB of individual cows. For example, during the first 8 WIM (across all parities), almost all observations (95.4%) with an FPR score > 1.5 were related to NEB (mean and S.D.; −56 ± 32.4 MJ of ME/day). However, during the same period, 79.2% of observations that had a ‘Normal’ FPR (between 1.0 and 1.5) were also in NEB (−33.3 ± 23.4 MJ of ME/day). Therefore, the large overlap in EB values between these two FPR score categories (1.0–1.5, and >1.5) confirms that FPR alone cannot accurately predict EB of individual cows. Furthermore, between 9 and 16 WIM, the majority of cows had an FPR score of between 1 and 1.5, while 47.9% of the observations with this score remained in NEB (−18 ± 16.6 MJ of ME/day). This supports the findings of previous studies that FPR had a limited predictive power and lacks precision to estimate the EB of individual cows [18,25,67]. Rather, FPR may be a more useful tool at a herd level, providing information on the general energy status of a group of cows [25,74]. Novel approaches such as the definition of metabolic clusters [75,76,77,78] could be useful for characterizing individuals on the basis of their metabolic efficiency.

## 5. Conclusions

Results obtained in the present study provide a better understanding of the EB responses per unit of FPR in milk during early–mid lactation (1 to 20 WIM) in Holstein-Friesian cows offered grass silage-based diets. While FPR in milk may provide a useful indication of EB at a herd level, it does not provide a robust indicator of EB at an individual cow level. The use of ‘High’ FPR scores in conjunction with additional indicators of EB appears to be helpful when describing physiological changes encompassing EB profiles at specific stages of lactation, until cows achieve positive EB. Caution should be exercised when extrapolating results of the present study, since the value of FPR in milk as an indicator of EB during early lactation may differ considerably depending on experimental conditions.

## Figures and Tables

**Table 1:** Mean and S.D. of data contained within the dataset, arranged by lactation number, obtained from 27 experiments involving 840 individual Holstein-Friesian dairy cows during early lactation (1–20 WIM).

Item ^1^	Lactation 1			Lactation 2			Lactation 3			Lactation ≥ 4		
	*n*	Mean	S.D.	*n*	Mean	S.D.	*n*	Mean	S.D.	*n*	Mean	S.D.
**Individual lactations, *n***	474			405			252			190		
Intake												
Forage DM, kg/day	8873	7.7	2.14	7556	9.7	2.56	4612	10.5	2.64	4564	10.7	2.71
Concentrate DM, kg/day	8873	8.5	2.77	7556	10.3	3.30	4612	10.8	3.27	4564	11.2	3.18
Total DMI, kg/day	8873	16.3	3.09	7556	19.9	3.62	4612	21.3	3.83	4564	21.9	3.79
Total ME intake, MJ/day	8873	198	39.3	7556	242	47.1	4612	259	49.9	4564	268	48.1
**Milk yield and comp.**												
Milk, kg/day	8835	27.2	5.12	7503	34.4	6.57	4587	38.0	7.17	4537	39.4	8.36
ECM, kg/day	8833	27.7	4.89	7498	35.1	6.27	4577	38.6	7.02	4534	39.8	8.06
Fat, g/kg	8873	41.0	6.44	7556	41.3	6.62	4612	41.7	7.23	4563	41.3	7.12
Protein, g/kg	8873	33.0	2.91	7556	33.6	3.04	4612	33.3	3.05	4563	32.9	2.99
Lactose, g/kg	8873	48.8	2.09	7556	48.0	2.02	4612	47.4	2.09	4563	47.2	2.13
Milk E output, MJ of ME/day	8873	86.0	15.1	7498	109	19.4	4577	120	21.7	4528	124	24.9
FPR, ratio	8873	1.25	0.190	7556	1.23	0.185	4612	1.25	0.208	4563	1.26	0.202
**Body reserves**												
BW, kg	8730	517	46.8	7469	568	50.6	4534	611	57.0	4526	637	61.4
BCS, units	6520	2.57	0.237	6588	2.40	0.238	3947	2.38	0.295	4012	2.36	0.304
BW change ^2^, kg/wk.	8362	0.67	9.94	7178	1.00	9.87	4347	0.34	10.3	4366	−0.24	10.9
**Blood metabolites**												
BHB, mmol/L	3700	0.54	0.289	2723	0.56	0.317	1918	0.59	0.298	1745	0.59	0.344
NEFA, mEq/L	3697	0.43	0.333	2733	0.36	0.271	1916	0.38	0.292	1731	0.39	0.307
**Feed efficiency and EB**												
ECM/DMI ^3^, kg/kg	8833	1.74	0.361	7499	1.79	0.341	4577	1.86	0.405	4528	1.85	0.402
EB ^4^, MJ of ME/day	8707	−2.16	34.8	7415	−4.71	40.4	4506	−10.5	47.7	4487	−10.5	49.5

^1^ ECM—energy-corrected milk; FPR—fat-to-protein ratio in milk; BHB—beta-hydroxybutyrate; NEFA—non-esterified fatty acids. ^2^ BW change represents the difference between current and previous measurement at each week postpartum. ^3^ Feed efficiency. ^4^ EB—energy balance estimated according to equations within ‘Feed into Milk’ [36].

**Table 2:** Partial correlations coefficients between fat-to-protein ratio in milk (FPR) and daily energy balance (EB, MJ of ME/day) in Holstein-Friesian dairy cows during early lactation (1–20 WIM) ^1^.

WIM ^2^	Lactation 1			Lactation 2			Lactation 3			Lactation ≥ 4		
	*n*	r	*p*-Value	*n*	r	*p*-Value	*n*	r	*p*-Value	*n*	r	*p*-Value
1–4	1554	−0.411	<0.001	1359	−0.328	<0.001	814	−0.354	<0.001	802	−0.408	<0.001
5–8	1851	−0.311	<0.001	1585	−0.271	<0.001	986	−0.387	<0.001	970	−0.335	<0.001
9–12	1869	−0.257	<0.001	1579	−0.207	<0.001	957	−0.325	<0.001	953	−0.249	<0.001
13–16	1796	−0.165	<0.001	1525	−0.168	<0.001	931	−0.217	<0.001	932	−0.245	<0.001
17–20	1637	−0.118	<0.001	1367	−0.148	<0.001	818	−0.119	<0.001	830	−0.192	<0.001

^1^ Correlations were controlled for the effect of individual study within the dataset; ^2^ weeks in milk/ stage of lactation.

**Table 3:** Linear mixed regression models between fat-to-protein ratio in milk (FPR) and daily energy balance (EB, MJ of ME/day) in Holstein-Friesian dairy cows during early lactation (1–20 WIM), and comparisons of regression slopes between WIM intervals within each lactation.

Lactation	WIM ^1^	*n*	Intercept	s.e.	Slope ^2^	s.e.	*p*-Value	Adj. RMSE ^3^	*p*-Value for Difference in Slope (WIM) ^4^				
									1–4	5–8	9–12	13–16	17–20
1	1–4	1554	49.0	6.34	−54.6	3.73	<0.001	21.4	--	0.012	0.133	<0.001	<0.001
	5–8	1851	50.5	5.63	−47.5	3.09	<0.001	17.0		--	0.271	<0.001	<0.001
	9–12	1869	65.4	5.65	−50.3	3.37	<0.001	15.4			--	<0.001	<0.001
	13–16	1796	53.3	5.67	−38.1	3.53	<0.001	14.6				--	0.026
	17–20	1637	49.2	5.50	−32.3	3.85	<0.001	14.7					--
2	1–4	1359	39.1	7.21	−57.2	4.79	<0.001	24.4	--	0.053	>0.500	>0.500	<0.001
	5–8	1585	68.7	7.00	−64.4	4.66	<0.001	21.1		--	0.011	0.049	<0.001
	9–12	1579	70.5	6.71	−55.6	4.50	<0.001	19.3			--	>0.500	0.001
	13–16	1525	75.6	6.80	−57.5	4.62	<0.001	17.8				--	<0.001
	17–20	1367	59.1	7.79	−43.0	5.62	<0.001	18.6					--
3	1–4	814	46.4	10.4	−72.8	7.28	<0.001	28.1	--	>0.500	<0.001	0.027	<0.001
	5–8	986	69.6	7.86	−72.8	5.11	<0.001	23.8		--	<0.001	0.010	<0.001
	9–12	957	113.2	8.55	−92.1	6.04	<0.001	22.3			--	<0.001	<0.001
	13–16	931	79.9	8.76	−61.5	6.29	<0.001	20.1				--	0.001
	17–20	818	63.2	10.1	−44.4	7.37	<0.001	21.2					--
≥4	1–4	802	66.6	9.84	−86.2	6.54	<0.001	30.6	--	0.118	0.028	<0.001	<0.001
	5–8	970	78.8	9.57	−77.6	6.13	<0.001	26.2		--	0.455	0.015	<0.001
	9–12	953	90.1	9.84	−73.7	6.42	<0.001	25.1			--	0.106	<0.001
	13–16	932	85.4	9.09	−65.1	6.33	<0.001	23.2				--	0.039
	17–20	830	78.5	10.6	−53.1	7.40	<0.001	23.5					--

^1^ Weeks in milk/stage of lactation; ^2^ linear rate of decrease in EB (MJ of ME/day) per 0.1 units of FPR; ^3^ root mean square error adjusted by the random crossed effects of study and cow; ^4^ pairwise comparisons of regression slopes according to T-test. Models for first lactation cows resulted in better curve fitting (lower RMSE) compared to models for multiparous cows. While regression slopes differed between many of the four-week periods within each lactation, the trends were generally inconsistent. However, irrespective of parity, regression slopes showed a significant decreased between 1–4 and 17–20 WIM (*p* < 0.001), and between 13–16 and 17–20 WIM; *p* ≤ 0.039).

**Table 4:** Performance indicators and blood metabolites (adjusted least squares means: 4 wk intervals) of first lactation Holstein-Friesian dairy cows within three fat-to-protein ratio scores in milk during early lactation (1–20 WIM).

WIM/Item ^1^	*n*	FPR score ^2^			s.e.m	*p*-Value
		<1.0	1.0–1.5	>1.5		
**Weeks 1–4 of lactation**						
DMI, kg/day	1639	13.4 ^ba^	13.7 ^a^	13.0 ^b^	0.38	<0.001
ECM, kg/day	1603	22.0 ^c^	25.6 ^b^	27.4 ^a^	0.55	<0.001
Milk fat, g/kg	1639	34.0 ^c^	43.3 ^b^	54.1 ^a^	0.55	<0.001
Milk protein, g/kg	1639	36.9 ^a^	34.5 ^b^	33.4 ^c^	0.36	<0.001
BCS, units	1097	2.67	2.65	2.66	0.039	0.387
BW change ^3^, kg/wk.	1246	−2.72 ^a^	−5.32 ^a^	−9.67 ^b^	0.947	<0.001
lnBHB ^4^	845	0.38 ^b^	0.44 ^b^	0.55 ^a^	0.024	<0.001
lnNEFA ^4^	843	0.32 ^c^	0.39 ^b^	0.45 ^a^	0.029	0.001
EB ^5^, MJ of ME/day	1554	−6.89 ^a^	−19.3 ^b^	−39.4 ^c^	4.712	<0.001
**Weeks 5–8 of lactation**						
DMI, kg/day	1884	16.0 ^a^	15.9 ^a^	15.5 ^b^	0.36	0.021
ECM, kg/day	1881	26.4 ^c^	28.4 ^b^	30.3 ^a^	0.55	<0.001
Milk fat, g/kg	1884	32.1 ^c^	40.2 ^b^	49.1 ^a^	0.38	<0.001
Milk protein, g/kg	1884	32.5 ^a^	32.1 ^b^	31.3 ^c^	0.28	<0.001
BCS, units	1384	2.56	2.56	2.58	0.034	0.084
BW change, kg/wk.	1839	2.71 ^a^	1.45 ^a^	−2.16 ^b^	0.686	<0.001
lnBHB	1034	0.41 ^c^	0.46 ^b^	0.52 ^a^	0.040	<0.001
lnNEFA	1037	0.32 ^b^	0.34 ^b^	0.38 ^a^	0.030	0.009
EB, MJ of ME/day	1851	3.53 ^a^	−9.16 ^b^	−23.7 ^c^	4.44	<0.001
**Weeks 9–12 of lactation**						
DMI, kg/day	1889	17.1 ^a^	17.0 ^a^	16.1 ^b^	0.40	<0.001
ECM, kg/day	1889	26.5 ^c^	28.4 ^b^	29.5 ^a^	0.55	<0.001
Milk fat, g/kg	1889	32.9 ^c^	40.0 ^b^	47.5 ^a^	0.42	<0.001
Milk protein, g/kg	1889	33.3 ^a^	32.5 ^b^	31.5 ^c^	0.29	<0.001
BCS, units	1385	2.52	2.52	2.52	0.032	>0.500
BW change, kg/wk	1860	4.06 ^a^	2.19 ^b^	−0.55 ^c^	0.633	<0.001
lnBHB	881	0.38 ^b^	0.43 ^a^	0.43 ^a^	0.020	<0.001
lnNEFA	878	0.25	0.26	0.28	0.032	>0.500
EB, MJ of ME/day	1869	14.6 ^a^	3.74 ^b^	−13.6 ^c^	4.17	<0.001
**Weeks 13–16 of lactation**						
DMI, kg/day	1816	17.2 ^ba^	17.3 ^a^	16.8 ^b^	0.42	0.011
ECM, kg/day	1816	26.4 ^c^	28.2 ^b^	29.4 ^a^	0.54	<0.001
Milk fat, g/kg	1816	33.3 ^c^	40.5 ^b^	47.7 ^a^	0.43	<0.001
Milk protein, g/kg	1816	34.0 ^a^	33.3 ^b^	32.2 ^c^	0.30	<0.001
BCS, units	1358	2.51	2.51	2.51	0.030	>0.500
BW change, kg/wk	1789	3.08	2.12	0.40	0.661	0.101
lnBHB	563	0.35 ^b^	0.43 ^a^	0.46 ^a^	0.024	<0.001
lnNEFA	562	0.20	0.23	0.23	0.040	0.226
EB, MJ of ME/day	1796	14.1 ^a^	7.05 ^b^	−6.82 ^c^	4.07	<0.001
**Weeks 17–20 of lactation**						
DMI, kg/day	1645	17.3	17.5	17.0	0.42	0.051
ECM, kg/day	1644	26.5 ^c^	27.7 ^b^	28.8 ^a^	0.65	<0.001
Milk fat, g/kg	1645	34.7 ^c^	40.9 ^b^	48.8 ^a^	0.53	<0.001
Milk protein, g/kg	1645	34.5 ^a^	33.7 ^b^	32.5 ^c^	0.28	<0.001
BCS, units	1296	2.53 ^a^	2.53 ^a^	2.48 ^b^	0.026	0.017
BW change, kg/wk.	1628	2.45	1.74	−0.23	0.711	0.136
lnBHB	377	0.38 ^b^	0.48 ^a^	0.48 ^a^	0.064	<0.001
lnNEFA	377	0.21	0.19	0.22	0.040	0.481
EB, MJ of ME/day	1637	14.3 ^a^	10.2 ^a^	−0.92 ^b^	3.461	<0.001

^a,b,c^ Means within a row with different superscripts differ (*p* ≤ 0.05) within FPR scores; ^1^ weeks in milk/ stage of lactation; ECM—Energy-corrected milk; FPR—fat-to-protein ratio; ^2^ FPR score—fat-to-protein ratio in milk; <1.0 = ‘Low’; 1.0–1.5 = ‘Normal’; >1.5 = ‘High’; ^3^ BW change represents the difference between current and previous BW at each week postpartum; ^4^ values for blood metabolites were natural log transformed; ^5^ EB—energy balance estimated according to equations within ‘Feed into Milk’ [36].

**Table 5:** Performance indicators and blood metabolites (adjusted least squares means: 4 wk intervals) of second lactation Holstein-Friesian dairy cows within three fat-to-protein ratio scores in milk during early lactation (1–20 WIM).

WIM/Item ^1^	*n*	FPR Score ^2^			s.e.m	*p*-Value
		<1.0	1.0–1.5	>1.5		
**Weeks 1–4 of lactation**						
DMI, kg/day	1417	17.2	17.2	16.9	0.46	0.450
ECM, kg/day	1382	30.7 ^c^	34.7 ^b^	37.0 ^a^	0.79	<0.001
Milk fat, g/kg	1417	36.1 ^c^	44.1 ^b^	55.1 ^a^	0.47	<0.001
Milk protein, g/kg	1417	37.8 ^a^	35.3 ^b^	34.2 ^c^	0.31	<0.001
BCS, units	1162	2.43 ^b^	2.44 ^b^	2.48 ^a^	0.021	0.016
BW change ^3^, kg/wk.	1124	−0.46 ^a^	−3.39 ^a^	−6.56 ^b^	1.097	<0.001
lnBHB ^4^	674	0.46 ^b^	0.49 ^b^	0.56 ^a^	0.026	<0.001
lnNEFA ^4^	674	0.34 ^b^	0.35 ^b^	0.40 ^a^	0.026	0.037
EB ^5^, MJ of ME/day	1359	−11.5 ^a^	−33.4 ^b^	−51.2 ^c^	4.63	<0.001
**Weeks 5–8 of lactation**						
DMI, kg/day	1611	20.3	19.9	19.7	0.44	0.085
ECM, kg/day	1604	33.8 ^c^	36.6 ^b^	38.9 ^a^	0.78	<0.001
Milk fat, g/kg	1611	33.3 ^c^	40.3 ^b^	47.7 ^a^	0.42	<0.001
Milk protein, g/kg	1611	32.7 ^a^	32.2 ^b^	31.6 ^c^	0.18	<0.001
BCS, units	1411	2.36	2.37	2.37	0.020	>0.500
BW change, kg/wk.	1584	3.93 ^a^	1.88 ^ba^	0.23 ^b^	0.694	0.013
lnBHB	833	0.43 ^b^	0.46 ^b^	0.52 ^a^	0.031	<0.001
lnNEFA	846	0.21 ^c^	0.26 ^b^	0.31 ^a^	0.023	<0.001
EB, MJ of ME/day	1585	8.08 ^a^	−12.4 ^b^	−29.3 ^c^	4.62	<0.001
**Weeks 9–12 of lactation**						
DMI, kg/day	1595	20.9 ^a^	20.7 ^a^	20.1 ^b^	0.47	0.004
ECM, kg/day	1593	33.5 ^c^	35.7 ^b^	37.8 ^a^	0.82	<0.001
Milk fat, g/kg	1595	33.0 ^c^	40.4 ^b^	48.0 ^a^	0.46	<0.001
Milk protein, g/kg	1595	33.2 ^a^	32.7 ^b^	31.6 ^c^	0.21	<0.001
BCS, units	1413	2.37	2.37	2.36	0.019	>0.500
BW change, kg/wk.	1572	3.09 ^a^	2.09 ^a^	−0.71 ^b^	0.682	0.014
lnBHB	637	0.38 ^b^	0.43 ^a^	0.48 ^a^	0.022	<0.001
lnNEFA	634	0.23	0.21	0.22	0.027	0.498
EB, MJ of ME/day	1579	17.6 ^a^	1.70 ^b^	−18.4 ^c^	4.45	<0.001
**Weeks 13–16 of lactation**						
DMI, kg/day	1547	20.7	20.7	20.6	0.51	>0.500
ECM, kg/day	1541	32.3 ^c^	34.6 ^b^	37.5 ^a^	0.80	<0.001
Milk fat, g/kg	1547	33.7 ^c^	40.8 ^b^	49.8 ^a^	0.49	<0.001
Milk protein, g/kg	1547	34.1 ^a^	33.5 ^b^	32.7 ^c^	0.21	<0.001
BCS, units	1356	2.39	2.38	2.39	0.020	>0.500
BW change, kg/wk.	1527	1.96	1.60	1.90	0.712	>0.500
lnBHB	363	0.34 ^b^	0.42 ^a^	0.44 ^a^	0.030	<0.001
lnNEFA	364	0.21	0.19	0.23	0.036	0.164
EB, MJ of ME/day	1525	18.9 ^a^	5.31 ^b^	−8.86 ^b^	4.68	<0.001
**Weeks 17–20 of lactation**						
DMI, kg/day	1386	20.5	20.2	20.3	0.54	>0.500
ECM, kg/day	1378	32.1 ^c^	33.4 ^b^	35.3 ^a^	0.90	<0.001
Milk fat, g/kg	1386	35.5 ^c^	41.3 ^b^	50.8 ^a^	0.53	<0.001
Milk protein, g/kg	1386	35.1 ^a^	34.1 ^b^	33.5 ^c^	0.21	<0.001
BCS, units	1246	2.43	2.42	2.41	0.018	>0.500
BW change, kg/wk.	1371	3.01	1.58	0.30	0.840	0.141
lnBHB	216	0.40 ^b^	0.46 ^a^	0.49 ^a^	0.060	0.008
lnNEFA	215	0.20	0.17	0.23	0.036	0.254
EB, MJ of ME/day	1367	16.5 ^a^	6.84 ^b^	−5.00 ^c^	4.63	<0.001

^a,b,c^ Means within a row with different superscripts differ (*p* ≤ 0.05) within FPR scores; ^1^ weeks in milk/ stage of lactation; ECM—energy corrected milk; FPR—fat-to-protein ratio; ^2^ FPR score—fat-to-protein ratio in milk; <1.0 = ‘Low’; 1.0–1.5= ‘Normal’; >1.5 = ‘High’; ^3^ BW change represents the difference between current and previous BW at each week postpartum; ^4^ values for blood metabolites were natural log transformed; ^5^ EB—energy balance estimated according to equations within ‘Feed into Milk’ [36].

**Table 6:** Performance indicators and blood metabolites (adjusted least squares means: 4 wk intervals) of third lactation Holstein-Friesian dairy cows within three fat-to-protein ratio scores in milk during early lactation (1–20 WIM).

WIM/Item ^1^	*n*	FPR Score ^2^			s.e.m	*p*-Value
		<1.0	1.0–1.5	>1.5		
**Weeks 1–4 of lactation**						
DMI, kg/day	870	17.8 ^ba^	18.3 ^a^	17.5 ^b^	0.49	0.018
ECM, kg/day	849	35.1 ^c^	38.7 ^b^	40.8 ^a^	0.87	<0.001
Milk fat, g/kg	870	35.7 ^c^	44.1 ^b^	54.2 ^a^	0.58	<0.001
Milk protein, g/kg	870	36.9 ^a^	35.1 ^b^	33.9 ^c^	0.41	<0.001
BCS, units	693	2.41	2.45	2.45	0.032	0.399
BW change ^3^, kg/wk.	668	−2.29 ^a^	−5.07 ^a^	−9.13 ^b^	1.459	0.004
lnBHB ^4^	510	0.48 ^b^	0.49 ^b^	0.60 ^a^	0.030	<0.001
lnNEFA ^4^	509	0.44 ^b^	0.53 ^b^	0.68 ^a^	0.058	<0.001
EB ^5^, MJ of ME/day	814	−29.9 ^a^	−45.4 ^b^	−66.3 ^c^	5.90	<0.001
**Weeks 5–8 of lactation**						
DMI, kg/day	1003	21.1 ^ba^	21.2 ^a^	20.4 ^b^	0.49	<0.001
ECM, kg/day	999	36.6 ^c^	40.6 ^b^	44.0 ^a^	0.81	<0.001
Milk fat, g/kg	1003	31.4 ^c^	40.1 ^b^	49.4 ^a^	0.42	<0.001
Milk protein, g/kg	1003	32.2 ^a^	32.1 ^a^	30.9 ^b^	0.20	<0.001
BCS, units	867	2.35	2.34	2.34	0.032	>0.500
BW change, kg/wk.	982	2.90 ^a^	2.26 ^a^	−2.20 ^b^	0.753	<0.001
lnBHB	624	0.44 ^b^	0.45 ^b^	0.52 ^a^	0.026	<0.001
lnNEFA	625	0.37 ^ba^	0.34 ^b^	0.46 ^a^	0.035	<0.001
EB, MJ of ME/day	986	0.64 ^a^	−20.7 ^b^	−52.9 ^c^	5.27	<0.001
**Weeks 9–12 of lactation**						
DMI, kg/day	972	22.8 ^a^	22.0 ^b^	21.4 ^b^	0.51	<0.001
ECM, kg/day	969	35.2 ^c^	38.6 ^b^	42.0 ^a^	0.82	<0.001
Milk fat, g/kg	972	32.1 ^c^	40.4 ^b^	48.5 ^a^	0.48	<0.001
Milk protein, g/kg	972	33.4 ^a^	32.7 ^b^	32.0 ^c^	0.26	<0.001
BCS, units	832	2.32	2.33	2.33	0.030	>0.500
BW change, kg/wk.	951	4.21 ^a^	1.32 ^b^	−1.32 ^b^	0.833	0.001
lnBHB	445	0.40	0.43	0.46	0.021	0.072
lnNEFA	444	0.21 ^ba^	0.21 ^b^	0.27 ^a^	0.024	0.054
EB, MJ of ME/day	957	30.1 ^a^	−1.37 ^b^	−26.6 ^c^	4.98	<0.001
**Weeks 13–16 of lactation**						
DMI, kg/day	941	22.3 ^a^	21.8 ^ba^	21.3 ^b^	0.54	0.044
ECM, kg/day	937	35.1 ^c^	36.9 ^b^	39.3 ^a^	0.93	<0.001
Milk fat, g/kg	941	33.5 ^c^	40.8 ^b^	50.1 ^a^	0.52	<0.001
Milk protein, g/kg	941	34.3 ^a^	33.4 ^b^	32.3 ^c^	0.26	<0.001
BCS, units	811	2.33	2.35	2.34	0.030	>0.500
BW change, kg/wk.	928	2.96 ^a^	1.73 ^a^	−2.95 ^b^	0.999	0.004
lnBHB	220	0.42	0.44	0.48	0.041	0.252
lnNEFA	220	0.17	0.20	0.24	0.035	0.077
EB, MJ of ME/day	931	23.0 ^a^	4.55 ^b^	−15.7 ^c^	5.26	<0.001
**Weeks 17–20 of lactation**						
DMI, kg/day	826	21.3	21.6	21.0	0.56	0.099
ECM, kg/day	823	33.6 ^c^	35.4 ^b^	37.8 ^a^	0.82	<0.001
Milk fat, g/kg	826	35.2 ^c^	41.7 ^b^	49.9 ^a^	0.60	<0.001
Milk protein, g/kg	826	34.3 ^a^	33.9 ^a^	33.5 ^b^	0.26	0.002
BCS, units	744	2.41 ^a^	2.39 ^ba^	2.35 ^b^	0.030	0.039
BW change, kg/wk.	818	1.72	1.07	−0.63	0.816	0.293
lnBHB	119	0.44	0.45	0.53	0.077	0.131
lnNEFA	118	0.19	0.17	0.18	0.036	>0.500
EB, MJ of ME/day	818	20.4 ^a^	9.02 ^b^	−10.3 ^c^	5.40	<0.001

^a,b,c^ Means within a row with different superscripts differ (*p* ≤ 0.05) within FPR scores; ^1^ weeks in milk/ stage of lactation; ECM—energy corrected milk; FP—fat-to-protein ratio; ^2^ FPR score—fat-to-protein ratio in milk; <1.0= ‘Low’; 1.0–1.5 = ‘Normal’; >1.5 = ‘High’; ^3^ BW change represents the difference between current and previous BW at each week postpartum; ^4^ values for blood metabolites were natural log transformed; ^5^ EB—energy balance estimated according to equations within ‘Feed into Milk’ [36].

**Table 7:** Performance indicators and blood metabolites (adjusted least squares means: 4 wk intervals) of ≥ fourth lactation Holstein-Friesian dairy cows within three fat-to-protein ratio scores in milk during early lactation (1–20 WIM).

WIM/Item ^1^	*n*	FPR Score ^2^			s.e.m	*p*-Value
		<1.0	1.0–1.5	>1.5		
**Weeks 1–4 of lactation**						
DMI, kg/day	848	19.1 ^a^	18.9 ^a^	17.7 ^b^	0.49	<0.001
ECM, kg/day	829	34.8 ^c^	39.2 ^b^	41.9 ^a^	1.21	<0.001
Milk fat, g/kg	848	34.9 ^c^	44.3 ^b^	55.1 ^a^	0.57	<0.001
Milk protein, g/kg	848	37.0 ^a^	35.1 ^b^	33.6 ^c^	0.33	<0.001
BCS, units	695	2.46	2.45	2.49	0.045	0.249
BW change ^3^, kg/wk.	669	−1.76 ^a^	−5.80 ^a^	−10.8 ^b^	1.583	<0.001
lnBHB ^4^	437	0.46 ^b^	0.51 ^b^	0.60 ^a^	0.032	<0.001
lnNEFA ^4^	434	0.36 ^b^	0.41 ^b^	0.49 ^a^	0.038	<0.001
EB ^5^, MJ of ME/day	802	−14.2 ^a^	−42.2 ^b^	−75.9 ^c^	5.99	<0.001
**Weeks 5–8 of lactation**						
DMI, kg/day	976	22.0 ^a^	21.7 ^a^	21.2 ^b^	0.54	0.037
ECM, kg/day	972	37.4 ^c^	41.3 ^b^	43.7 ^a^	1.28	<0.001
Milk fat, g/kg	976	32.1 ^c^	40.5 ^b^	48.6 ^a^	0.51	<0.001
Milk protein, g/kg	976	32.3 ^a^	31.9 ^a^	31.1 ^b^	0.28	<0.001
BCS, units	856	2.36	2.34	2.38	0.032	0.057
BW change, kg/wk.	973	3.07 ^a^	1.06 ^a^	−2.62 ^b^	0.905	<0.001
lnBHB	541	0.39 ^c^	0.47 ^b^	0.59 ^a^	0.033	<0.001
lnNEFA	539	0.26 ^b^	0.29 ^b^	0.37 ^a^	0.029	<0.001
EB, MJ of ME/day	970	5.06 ^a^	−20.9 ^b^	−41.9 ^c^	6.01	<0.001
**Weeks 9–12 of lactation**						
DMI, kg/day	964	22.6	22.5	22.2	0.52	0.366
ECM, kg/day	958	36.0 ^c^	39.6 ^b^	42.3 ^a^	1.31	<0.001
Milk fat, g/kg	963	31.8 ^c^	40.1 ^b^	49.1 ^a^	0.57	<0.001
Milk protein, g/kg	963	32.5 ^a^	32.3 ^a^	31.7 ^b^	0.30	0.004
BCS, units	858	2.32	2.31	2.31	0.035	>0.500
BW change, kg/wk.	956	2.45	1.85	0.74	0.852	>0.500
lnBHB	429	0.40 ^c^	0.44 ^ba^	0.49 ^a^	0.026	0.025
lnNEFA	423	0.19	0.22	0.25	0.023	0.096
EB, MJ of ME/day	953	18.4 ^a^	−2.04 ^b^	−21.8 ^c^	6.17	<0.001
**Weeks 13–16 of lactation**						
DMI, kg/day	942	22.9 ^a^	22.4 ^ba^	21.8 ^b^	0.55	0.038
ECM, kg/day	937	35.7 ^c^	37.9 ^b^	40.5 ^a^	1.23	<0.001
Milk fat, g/kg	942	32.9 ^c^	40.7 ^b^	49.6 ^a^	0.58	<0.001
Milk protein, g/kg	942	33.2 ^a^	33.0 ^a^	32.3 ^b^	0.29	0.009
BCS, units	841	2.30	2.33	2.28	0.039	0.083
BW change, kg/wk.	936	1.66 ^a^	1.14 ^a^	−3.03 ^b^	0.972	0.008
lnBHB	204	0.37 ^b^	0.43 ^a^	0.50 ^a^	0.032	0.006
lnNEFA	203	0.21	0.21	0.23	0.037	>0.500
EB, MJ of ME/day	932	22.4 ^a^	4.83 ^b^	−13.5 ^c^	5.68	<0.001
**Weeks 17–20 of lactation**						
DMI, kg/day	834	22.4	22.1	22.1	0.50	0.414
ECM, kg/day	832	33.4 ^c^	35.7 ^b^	37.6 ^a^	1.32	<0.001
Milk fat, g/kg	834	34.0 ^c^	41.1 ^b^	50.3 ^a^	0.63	<0.001
Milk protein, g/kg	834	33.7 ^a^	33.3 ^b^	32.2 ^c^	0.30	<0.001
BCS, units	762	2.40 ^a^	2.35 ^b^	2.26 ^c^	0.046	0.001
BW change, kg/wk.	831	2.32	0.54	−0.13	0.907	0.178
lnBHB	134	0.37	0.41	0.51	0.035	0.054
lnNEFA	132	0.19	0.18	0.28	0.038	0.128
EB, MJ of ME/day	830	29.6 ^a^	11.9 ^b^	2.25 ^b^	6.11	<0.001

^a,b,c^ Means within a row with different superscripts differ (*p* ≤ 0.05) within FPR scores; ^1^ weeks in milk/ stage of lactation; ECM—energy corrected milk; FPR—fat-to-protein ratio; ^2^ FPR score—fat-to-protein ratio in milk; <1.0= ‘Low’; 1.0–1.5= ‘Normal’; >1.5= ‘High’; ^3^ BW change represents the difference between current and previous BW at each week postpartum; ^4^ values for blood metabolites were natural log transformed; ^5^ EB—energy balance estimated according to equations within ‘Feed into Milk’ [36].

## Data Availability

The data presented in this paper are not publicly available due to confidentiality restrictions.

## Appendix A

List of publications (and experiment ID code) describing the experiments from which individual animal data was obtained (all experiments undertaken at the Agri-Food and Biosciences Institute, UK, between 1996 and 2016).

1. Ferris, C.P.; Gordon, F.J.; Patterson, D.C.; Mayne, C.S.; McCoy, M.A. A short-term comparison of the performance of four grassland-based systems of milk production for autumn-calving dairy cows. *Grass Forage Sci.* **2003**, *58*, 192–209. https://doi.org/10.1046/j.1365-2494.2003.00371.x **AFBI experiment codes C18 and C24.**
2. Ferris, C.P.; Patterson, D.C.; McCoy, M.A., Kilpatrick, D.J. Effect of offering dairy cows diets differing in phosphorus concentration over four successive lactations: 1. Food intake, milk production, tissue changes and blood metabolites. *Animal*. **2010**, *4*, 545–559. https://doi.org/10.1017/S1751731109990929 **AFBI experiment codes C36, C40, C42, and C48.**
3. Ferris, C.P.; Doody, D.G.; Laughlin, R.; Watson, C.J.; Watson, S. Cow performance within contrasting milk production systems over three successive lactations. In *Proceedings of British Society of Animal Science, 11th Research Conference: Science and Practice for Grass-Based Systems.* Dumfries, Scotland. 2003, Paper 6.3. **AFBI experiment codes C54, D91, and D100.**
4. Ferris, C.P.; Gordon, F.J.; Patterson, D.C.; Murphy, J. A three-year comparison of four contrasting grassland based systems of milk production. In *Profitable grass and forage: meeting the needs of the farmer and society. Proceedings of British Society of Animal Science,* Stafford, UK, 2002. **AFBI experiment codes C27 and C30.**
5. Gilmore, H.; Young, F.J.; Patterson, D.C.; Wylie, A.; Law, R.A.; Elliot, C.; Mayne, C.S. An evaluation of the effect of altering nutrition and nutritional strategies in early lactation on reproductive performance and oestrous behavior of high yielding Holstein-Friesian dairy cows. *J. Dairy Sci*. **2011**, *94*, 3510–3526. https://doi.org/10.3168/jds.2010-3547 **AFBI experiment code D80**.
6. Gilmore, H.S. An investigation of factors affecting reproductive function in the high genetic merit Holstein-Friesian dairy cow. *PhD Thesis*, School of Biological Sci., Queen’s University of Belfast. 2010. **AFBI experiment code D84.**
7. Gordon, F.J.; Ferris, C.P.; Patterson, D.C.; Mayne, C.S. A comparison of two grassland-based systems for autumn-calving dairy cows of high genetic merit. *Grass Forage Sci.* **2000**, *55*, 83–96. https://doi.org/10.1017/S1752756200002313 **AFBI experiment code C8.**
8. Johnston, D.J.; Theodoridou, K.; Gordon, A.W.; Yan T.; McRoberts, W.C.; Ferris C.P. Field bean inclusion in the diet of early lactation dairy cows: Effect on performance and nutrient utilization. *J. Dairy Sci.* **2019**, *102*, 10887–10902. https://doi.org/10.3168/jds.2019-16513 **AFBI experiment code D127.**
9. Keady, T.W.J.; Mayne, C.S. The effect of two levels of nutrient intake on milk production of two dairy cow genotypes. In *Proceedings of British Society of Animal Science,* 2002, pp. 12–12. doi:10.1017/S1752756200006682 **AFBI experiment code D40 (Yr. 2).**
10. Keady, T.W.J.; Mayne, C.S. An evaluation of the effect of concentrate proportion of the diet during previous and present lactations on animal performance of two breeds of lactating dairy cows. In *Proceedings of British Society of Animal Science*, 2003, pp. 24–24. doi:10.1017/S1752756200011832 **AFBI experiment code D40 (Yr. 3).**
11. Law, R.A.; Young, F.J.; Patterson, D.C.; Kilpatrick, D.J.; Wylie, A.R.G.; Ingvarsten, K.L.; Hameleers, A.; McCoy, M.A.; Mayne, C.S.; Ferris, C.P. Effect of pre calving and post calving dietary energy level on performance and blood metabolite concentrations of dairy cows throughout lactation. *J. Dairy Sci.* **2011**, *94*, 808–823. https://doi.org/10.3168/jds.2009-2728 **AFBI experiment code D64.**
12. Law, R.A., Young, F.J.; Patterson, D.C.; Kilpatrick, D.J.; Wylie, A.R.G.; Mayne, C.S. Effect of dietary protein content on animal production and blood metabolites of dairy cows during lactation. *J. Dairy Sci.* **2009**, *92*, 1001–1012. https://doi.org/10.3168/jds.2008-1155 **AFBI experiment code D71.**
13. Law, R.A.; McGettrick, S.A.; Ferris, C.P. Effect of three different concentrate build-up strategies in early lactation on production performance, health, and fertility of high yielding dairy cows. *End of project report for AgriSearch,* D-54-11. 2012. Access. Sep. 03, 2020 https://www.agrisearch.org/dairy/completed-dairy/feeding-dairy-completed/125-effect-of-three-different-concentrate-build-up-strategies-in-early-lactation-on-production-performance-health-and-fertility-of-high-yielding-dairy-cows **AFBI experiment code D97**
14. Law, R.A.; McGettrick, S.; Ferris, C.P. 2011. Effect of concentrate build-up strategy in early lactation on production performance, health and fertility of high-yielding dairy cows. In: Advances in Animal Biosciences-Food Security-Challenges and Opportunities for Animal Science. In *Proceedings of British Society of Animal Science and the Association of Veterinary Teaching and Research Work*, 2011, pp. 005–005. **AFBI experiment code D90.**
15. Purcell, P.J.; Law, R.A.; Gordon, A.W.; McGettrick, S.A.; Ferris, C.P. Effect of concentrate feeding method on the performance of dairy cows in early to mid-lactation. *J. Dairy Sci.* **2016**, *99*, 2811–2824. https://doi.org/10.3168/jds.2015-9988 **AFBI experiment code D102.**
16. Purcell, P.J.; Law, R.A.; Ferris, C.P. 2015. Effect of concentrate feed rate within a feed-to-yield system on the performance of dairy cows in early to mid-lactation. In *Advances in Animal Science–Science with Impact. Proceedings of British Society of Animal Science*.; Vol 6, Part 2, Cambridge University Press, 2015, pp. 192–192. **AFBI experiment code D107.**
17. Vance, E.R.; Ferris, C.P.; Elliot, C.T.; Hartley, H.M.; Kilpatrick, D.J. Comparison of the performance of Holstein-Friesian and Jersey x Holstein-Friesian crossbred dairy cows within three contrasting grassland-based systems of milk production. *Livest. Sci.* **2013**, *151*, 66–79. https://doi.org/10.1016/j.livsci.2012.10.011 **AFBI experiment code C49 and C51.**
18. Vance, E.R.; Ferris, C.P.; Elliot, C.T.; McGettrick, S.A.; Kilpatrick, D.J. Food intake, milk production, and tissue changes of Holstein-Friesian and Jersey × Holstein-Friesian dairy cows within a medium-input grazing system and a high-input total confinement system. *J. Dairy Sci.* **2012**, *95*, 1527–1544. https://doi.org/10.3168/jds.2011-4410 **AFBI experiment code C54 (V).**
19. Young, F.J.; Hameleers, A.; Patterson, D.C.; Mayne, C.S. 2006. Effect of additional concentrate supplementation for dairy cows at three time points in lactation. In *Proceedings of British Society of Animal Science*, 2006, p. 123. doi:10.1017/S1752756200018007 **AFBI experiment code D56**.
